# Developing a Punjab Index of Multiple Deprivation to investigate regional health inequalities in North-Western India

**DOI:** 10.1186/s12889-025-26073-x

**Published:** 2026-01-08

**Authors:** Sujata Sujata, Ramna Thakur, Martin Siegel

**Affiliations:** 1 School of Humanities and Social Sciences, Indian Institute of Technology Mandi, Mandi, Kamand Campus, Himachal Pradesh, 175075 India; 2 Heidelberg Institute of Global Health, Universitätsklinikum Heidelberg, Heidelberg University, Heidelberg, Germany; 3 Department of Empirical Health Economics, Technische Universität Berlin, Berlin, Germany; 4 Chair of General Economics, Health Economics and Econometrics, University of Greifswald, Greifswald, Germany

**Keywords:** Area deprivation, Diabetes, Obesity, Health inequality, Concentration index, India

## Abstract

**Background:**

Area deprivation indices are effective tools in health inequality research to identify social disparities and variations in health risks between different areas. We developed an index of multiple area deprivation for the Indian state of Punjab at the district level and present an application to inequalities in diabetes, general obesity (based on BMI), and abdominal obesity with respect to area deprivation and compare the results with household-level wealth-related inequalities.

**Methods:**

We used data from multiple sources and applied factor analysis to derive the Punjab Index of Multiple Deprivation (PIMD). Information on outcome variables and households’ socio-economic status was obtained from the 5th round of the National Family Health Survey (NFHS-5), conducted in 2019–21. It included 69,109 individuals from Punjab aged 15 and above. Deprivation-related and wealth-related inequalities in diabetes, general obesity and abdominal obesity were measured using the concentration index.

**Results:**

The overall prevalence is 6.13% for diabetes, 39.33% for general obesity and 58.26% for abdominal obesity in Punjab. Our results suggest a significant concentration of diabetes (C = 0.1595), general obesity (C = 0.1187) and abdominal obesity (C = 0.0755) by wealth among richer individuals and a significant concentration of diabetes (C = 0.0526), general obesity (C= 0.053) and abdominal obesity (C=0.0275) by deprivation in better-off areas.

**Conclusion:**

Area deprivation is an important determinant of the socio-economic gradients in diabetes and obesity. Policymakers should address the regionally unequal burden when allocating resources to prevent or mitigate the spread of diabetes in India.

**Supplementary Information:**

The online version contains supplementary material available at 10.1186/s12889-025-26073-x.

## Introduction

Individual and area-level socio-economic conditions affect access to essential goods and services that promote physical and psychological well-being, and ultimately impact health equity [32, 33, 66]. While significant associations of neighborhood disadvantages or area deprivation with health are well-documented for high-income countries (HICs) [7, 20, 30, 31, 56, 58], this association has rarely been explored in low and middle-income countries (LMICs). This paper develops an Index of Multiple Deprivation for the State of Punjab, located in the North-Western region of India, at the district level, designed to address the specific circumstances faced in LMICs. The index is applied to investigate regional-level inequalities in diabetes and its two prominent risk factors, general obesity (based on BMI) and abdominal obesity, to demonstrate its validity and usefulness. We chose Punjab because it is one of India's richest states with a population of around 27.7 million, covering an area of 50,362 square kilometers. The state exhibits the country’s second-highest Disability Adjusted Life Years (DALYs) rate for diabetes, and related risk factors such as unhealthy diets, high blood pressure, high cholesterol, and being overweight are widespread in Punjab [22]. In addition, a recent paper ranked Punjab high in overall diabetes vulnerability using an index based on multiple state-level risk indicators [57].

Area deprivation indices are effective tools in health inequality research for identifying social disparities and variations in health risks across regions. In the context of HICs, various deprivation indices such as Townsend’s Overall Deprivation Index [61], Carstairs’ Deprivation Index [7], or Jarman’s Underprivileged Area Score [23] were constructed and used in health-related studies. Carstairs and Morris [7] investigated the presence of a spatial effect on health outcomes beyond the effect of social class and found that area characteristics significantly influence health indicators. Noble and colleagues constructed Indices of Multiple Deprivation for England [37], Scotland [40, Wales [38], and Northern Ireland [39]. Further, deprivation indices were also constructed and deployed in health inequality research in Germany [18, 30, 31, 56], Australia [1, 43], France [20, Italy [60], Sweden [58], and Spain [46], where significant association between health and deprivation levels were found.

To our knowledge, in the context of LMICs, the concept of multiple area deprivation was to date only applied to South Africa [3, 36], China [64, 65, 68, 69] and specific regions in India, such as rural India, Kolkata, and urban slums [5, 34, 42, 53]. The social and economic composition in LMICs differs considerably from that in high-income countries, and the deprivation domains of established deprivation indices for HICs may not be fully adequate to describe area deprivation in India as an LMIC. We altered the included domains and derived economic, infrastructure, health, healthcare, living environment and educational deprivation domains to adapt the index to the context of Punjab, following the essential principles described by Michael Noble, Wright, Smith, and Dibben [41]. We applied the new deprivation index to an analysis of inequalities in diabetes, general obesity and abdominal obesity associated with both area deprivation and household-level wealth.

This paper is organized as follows: The next section describes the construction of the Punjab Index of Multiple Deprivation (PIMD), including data sources, grouping of domains and computation of the overall scores. The third section describes the measurement of wealth and deprivation-related inequalities. Section 4 presents the results, which are discussed in Section 5, and Section 6 gives some concluding remarks.

## The punjab index of multiple deprivation

### Domains and indicators

Previous studies included a range of domains and indicators for studying the regional pattern of deprivation in India. For instance, Mishra [34] developed a neighborhood deprivation index for the Indian state of Kolkata by including eight indicators across three domains: environmental, economic and household factors. Nolan et al. [42] constructed the Basic Services Deprivation Score for the urban slums in India by incorporating data on 12 items, i.e. the source of drinking water, latrine facility, sewer infrastructure, solid waste disposal, drainage system, access to electricity, quality of road within the slum, roads getting waterlogged in monsoon, quality of approach road to the slum, approach road getting waterlogged in monsoon, distance to nearest government primary school, and distance to nearest health center. Basu and Das [5] studied the regional pattern of rural deprivation in India by incorporating 31 indicators across seven domains: housing condition, environment and amenities, assets availability, socioeconomic status, deprivation, health and gender.

Considering these previous studies and data availability, we defined six domains that represent specific types of deprivation. Each domain comprises at least two indicators, which were selected based on the criteria described by Michael Noble et al. [41]: Indicators should be domain-specific, measure that deprivation as directly as possible, and address the main aspects of the deprivation, which are not only conditions endured by a small fraction of people or regions.

The first domain is economic deprivation. We chose the fraction of below-poverty-line (BPL) households and unemployment status as indicators to measure economic deprivation, because they reflect the proportion of individuals at risk of financial hardships in a region. The second domain is the infrastructural deprivation domain, which comprises the proportion of the population without access to banking services as financial infrastructure, the fraction of villages without paved roads as road connectivity infrastructure, and the number of secondary education facilities per 10,000 people as educational infrastructure. The third domain gauges health deprivation and includes the nutritional status of the population and the infant mortality rate. The inclusion of a health domain is not new, as, for example, the deprivation indices for England, Wales, Northern Ireland, and Scotland [37–40] also included health deprivation domains. There is a concern among researchers regarding the potential endogeneity when using health-related indicators in health inequality research. However, Bradford, Allik, McMahon, and Brown [6] showed that the risk of endogeneity bias is minimal when the health domain is included. We argue that the health indicators used in this study are good indicators of the population health, yet generic enough to not cause endogeneity problems in epidemiological studies which do not directly address the chosen indicators.

The fourth domain measures healthcare deprivation, and our PIMD is the first deprivation index to address potential insufficiencies of healthcare resources as a relevant dimension of multiple area deprivation. The healthcare deprivation domain comprises the number of primary and community healthcare centers, and the ratio of doctors per number of doctors officially required in each district. The fifth domain is the living environment deprivation domain, which measures the actual living and housing conditions experienced by the population in their everyday life. It includes the type of cooking fuel used, the sources of drinking water and illumination, and the construction type of housing. For the construction type of housing, we distinguish houses where walls and roofs consist of raw materials that require frequent replacement, such as unburnt bricks, bamboo, mud, grass, reeds, thatch, plastic/polythene, loosely packed stone, and similar materials (referred to as kaccha houses in India) from solid houses built with durable materials (referred to as pucca houses in India). These indicators are eminently relevant when measuring deprivation in an LMIC, and the living environment deprivation may be the most distinct difference between our index and its predecessors derived for HICs.

Lastly, we included educational deprivation as our sixth domain. Educational deprivation domains are a common component of indices of multiple deprivation, but we adapted the set of indicators to the situation in India as an LMIC. In particular, we chose the children’s school attendance rate, the proportion of the population without complete primary education, and the female illiteracy rate. Table 1 shows the complete list of indicators and the type of measurements used.

**Table 1:** Domains and indicators

*Domains*	*Indicators*	*Data sources*
*Economic deprivation*	- percentage of households having a BPL card - unemployment rate	NFHS-4 (2015–16) Census 2011 [8]
*Infrastructural deprivation*	***-*** percentage of households with no access to banking services - number of schools up to higher secondary level per 10,000 population - percentage of villages without paved roads	Census 2011 [8] DISE 2016–17 Census 2011 [8]
*Health deprivation*	- infant (under one year) mortality rate per 1000 live births - fraction of malnourished population	Census 2011 [8] NFHS-4 (2015–16)
*Healthcare deprivation*	- number of community and primary health centers per 100,000 population - the ratio of doctors per the number of doctors required as per Indian Public Health Standards (IPHS) norms	Rural Health Statistics 2018–19 Best Practices in the Performance of District Hospitals 2021, NITI Aayog
*Living environment deprivation*	- percentage of households using unclean (solid) fuel - percentage of households drinking water mainly from untreated sources - percentage of households living in mud (kaccha) houses - percentage of households where electricity is not the main source of lighting	Census 2011 [8] Census 2011 [8] Census 2011 [8] Census 2011 [8]
*Educational deprivation*	- percentage of the population without a completed primary education - percentage of school-aged children not attending school - female illiteracy rate	NFHS-4 (2015–16) NFHS-4 (2015–16) Census 2011 [8]

The table lists the six domains with the included indicators. BPL: Below poverty line, NFHS-4: 4th round of National Family Health Survey, DISE: District Information System for Education

### Sources of regional-level data

Indicators used in deprivation indices must be up to date, and regular updates must be feasible. Indicators must also be statistically robust and provided at a consistent small-area level for the entire area to be studied [41]. Therefore, we chose variables from the Census of India (2011) [8], National Family Health Survey (NFHS) [35], District Information System for Education (DISE) [16], and Rural Health Statistics [48], which are updated periodically. We chose districts as our areal unit of analysis because they are the smallest unit for which all required data are available. One challenge was that Fazilka bifurcated from Firozpur and Pathankot bifurcated from Gurdaspur in July 2011, which increased the number of districts from 20 to 22. Since the most recent census data available at the time this study was conducted were collected and published before the two new districts were formed, we assigned the Census 2011 [8] information for Firozpur also to Fazilka, and the information for Gurdaspur also to Pathankot.

### Summary of indicators on domains

All indicators were z-standardized to remove differences in means and variations, such that all variables have a zero mean and a standard deviation equal to one in all the following computations. Principal Component Analysis (PCA) and Geographically Weighted Principal Component Analysis (GWPCA) were found to be the most commonly utilized methods to construct a composite index [5, 34, 51, 52]. However, it is important to acknowledge that when utilizing PCA or factor analysis-generated weights, several challenges need to be addressed. These challenges include variables being measured on different scales, variations in statistical accuracy, varying distributions, and imperfect measurement of the underlying factor. PCA, for example, does not fully account for these issues and tends to overlook error variance, treating common variance as the total variance. In cases where specific and error variances are suspected, a more appropriate technique is Maximum Likelihood (ML)-based factor analysis [41]. Additionally, previous studies did not take into account the different deprivation domains during the combination processes [36]. For example, for the index constructed by Basu and Das [5] 20 out of 31 indicators were related to just three domains, the remaining 11 to the four domains.

Hence, unlike factor analysis in much of the social sciences, we employed a one-common-factor model merely to reduce the dimensionality to one-dimensional domain scores. More specifically, we ran domain-specific maximum-likelihood-based factor analyses to obtain weights for each indicator within domains with more than two indicators [41]. Scores for domains with only two indicators were computed as the arithmetic mean, i.e., with equal weights of $0.5$.

To ensure a sufficiently large sample size for running the factor analysis [15, 50], we sampled an additional 66 districts from Tamil Nadu, Kerala, Goa, and Himachal Pradesh in the analysis, which increased the sample size to 88 districts. Alike the districts from Punjab, the additional districts fall into the highest Epidemiological Transitioning Levels (ETLs) group, and rank high in overall diabetes vulnerability [57] and also exhibit high all-age DALY rates for diabetes (details in the Supplementary Material 1) [14]. We included all the districts in the factor analysis, but the additional 66 districts from additional four states (Tamil Nadu, Kerala, Goa, and Himachal Pradesh) were not included in the following analyses.

### Combination of domains to PIMD

After obtaining the domain scores, we combined them into an overall index of multiple deprivation. For this, we ranked only the $A=22$ districts of Punjab by the scores for each of the six domains. The ranking was done in such an order that the lowest rank ${R}_{D}=\frac{1}{A}$ was assigned to the least deprived district in the respective domain $D$, and the highest rank ${R}_{D}=1$ was assigned to the most deprived district in the respective domain [41]. After that, the ranks were transformed using Eq. (1)

1 $${X}_{D}= -23\mathrm{ln}\left\{1-{R}_{D}\left[1-exp\left(-\frac{100}{23}\right)\right]\right\},$$

to achieve a right-skewed distribution of the domain scores ${X}_{D}$ within each domain $D$. The resulting domain scores ${X}_{D}$ range between 0 and 100, where a score of 100 is given to the most deprived district, and only the most deprived 10% of the districts receive domain scores ${X}_{D}>50$. This step prevents that high deprivation in one domain can easily be compensated by average or low deprivation in only one or two other domains when summarizing the domain scores into a univariate index of multiple deprivation. We assigned equal weights to all six domains and summarized them into an arithmetic mean to create the scores for the overall PIMD, and ranked the 22 districts by the resulting scores.

## Statistical analysis

### Individual-level survey data and variables

We used the 5th round of the National Family Health Survey (NFHS-5) data on Punjab, conducted in 2019–21, which includes 69,109 individuals from Punjab in the age group 15 and above. Pregnant women (n = 560) and observations with missing and flagged information on the outcome variable (*n* = 15,334) and age (*n* = 15) were excluded. The final dataset for analysis of diabetes included 52,682 adults, excluding those who did not sleep in the house the night before the survey (*n* = 518) [35]. District-wise distribution of the sample is given in the appendix. A notable characteristic of the NFHS is the discrepancy in sample sizes across health outcomes, with 52,682 observations for glucose levels, 22,161 for body mass index (BMI), and 22,273 for waist circumference. We refrain from complete case analysis due to the small overlap in sub-samples, which would have resulted in only 22,161 observations.

We considered a person to have diabetes if the non-fasting glucose level was $\ge$ 200 mg/dl, if the fasting glucose level was $\ge$ 126 mg/dl, or if the person reported being on medication to lower the glucose level [4, 12]. NFHS-5 conducted biomarker measurements with the help of trained health investigators in each team. Random blood glucose was measured using a finger-stick blood specimen for women and men aged 15 and above, using the Accu-check Performa Glucometer with glucose test strips [21].

Furthermore, following Asia–Pacific guidelines, the general obesity is measured through BMI (BMI $\ge$ 25) [19, 28], and abdominal obesity is defined as a waist circumference of more than 80 cm in women and more than 94 cm in men. We used waist circumference rather than waist-to-hip ratio because the former is considered a more accurate measure of abdominal obesity, as a low waist-to-hip ratio could be due to high hip circumference and vice versa [10]. We derived our outcome variables as binary indicators for diabetes, general obesity and abdominal obesity. We gauged a household’s socio-economic status using its household-level wealth index factor score, which is included in the NFHS-5 dataset [35, 49], as a surrogate. The wealth index score is derived using Principal Component analysis (PCA). The households were given scores based on the availability of several consumer goods and household characteristics such as source of water, toilet facilities, and flooring materials [35]. We used sampling weights in our analyses to account for non-proportional allocation, non-response, and random selection, ensuring an accurate representation of the population in the survey data.

### Wealth and deprivation-related health inequalities

To measure inequality, we employed the concentration index $C$, which summarizes the information from the concentration curve. The concentration curve plots the cumulative proportion of the health variable on the y-axis against the cumulative proportion of the population ranked by socio-economic status on the x-axis, beginning with the most disadvantaged and ending with the least disadvantaged. If the curve lies above (below) the 45-degree line, it indicates a disproportionate concentration of the outcome among the worse-off (better-off). C measures twice the area between the concentration curve and the line of equality, and ranges between $(-\mathrm{1,1})$.

In this study, C is calculated using the convenient regression approach suggested by Kakwani, Wagstaff, and Van Doorslaer [25] and Wagstaff, O'Donnell, Van Doorslaer, and Lindelow [63],

2 $$\frac{2{\sigma }_{r}^{2}}{\overline{y}}{y }_{i}={\beta }_{0}+{\beta }_{1}{r}_{i}+{\varepsilon }_{i},$$

where ${r}_{i}={\sum }_{j=1}^{i}{w}_{j}-\frac{{w}_{i}}{2}$ is the weighted fractional rank with variance ${\sigma }_{r}^{2}$ and individuals $i=1,\dots ,n$ are ranked in ascending order by the socio-economic status indicator, i.e., wealth index factor score and PIMD. $\overline{y }$ is the mean of the health variable y, and ${w}_{i}$ is the sample weight scaled to sum to 1, i.e., ${\sum }_{i=1}^{n}{w}_{i}=1$. Estimating the parameters ${\beta }_{0}$ and ${\beta }_{1}$ of the linear regression model gives ${\beta }_{1}$ as the value of the concentration index C. Both, wealth-related and deprivation-related inequalities, are estimated separately using the same sample of individuals and incorporating the sample weights.

### Sensitivity to the choice of the socio-economic status variable

We further investigated how the choice of the socio-economic indicator, household-level wealth or district-level PIMD, affects the measured degree of inequality [29]. For this, we computed the difference between the concentration indices ${C}_{1}$ and ${C}_{2}$, where ${C}_{1}$ measures the wealth-related inequality and ${C}_{2}$ measures the deprivation-related inequality. Denoting ${r}_{1}$ the fractional rank with respect to the wealth index factor score, and ${r}_{2}$ the fractional rank with respect to the district’s PIMD, and $\Delta {r}_{i}={r}_{2}-{r}_{1}$, the difference in inequalities $\Delta C={C}_{2}-{C}_{1}$ can be written as

3 $$\Delta C=\frac{2}{n\overline{y} }\sum_{i=1}^{n}{y}_{i}\Delta {r}_{i}$$

[29, 56] and can be estimated through the convenient regression approach in Eq. (2). The difference between these two indices, $\Delta C={C}_{2}-{C}_{1},$ represents rank sensitivity, the change in the degree of inequality when shifting from an individual-level measure of socioeconomic status to an area-level measure. A positive $\Delta C$ indicates that inequality is more pronounced when assessed with district-level deprivation. In contrast, a negative $\Delta C$ suggests greater inequality when using household-level wealth as the ranking variable [29].

### Statistical inference

One advantage of the convenient regression approach is that it allows us to estimate the standard error of $C$ Wagstaff, O'Donnell, Van Doorslaer, and Lindelow [63]. Estimating the standard error of ${\widehat{\beta }}_{1}$ obtained from the regression in Eq. (2) might be imprecise because of the sampling variability of $\overline{y }$. We therefore estimated the standard errors of the coefficients ${\widetilde{\beta }}_{0}$ and ${\widetilde{\beta }}_{1}$ from a linear regression with untransformed outcome and coefficients, ${y}_{i}={\widetilde{\beta }}_{0}+{\widetilde{\beta }}_{1}{r}_{i}+{\epsilon }_{i}^{*}$, and approximated the standard errors ${\sigma }_{C}$ and ${\sigma }_{\Delta C}$ of C and $\Delta C$ by applying Rao's delta method to the transformation formula $C= \frac{2{\sigma }_{r}^{2}}{{\widetilde{\beta }}_{0}+\overline{r}{\widetilde{\beta } }_{1}}{\widetilde{\beta }}_{1}$, with $\overline{r }$ as arithmetic mean of the rank variable [25, 29, 45, 56, 63, 67]. Some authors expressed concerns regarding the accuracy of the covariance matrix obtained from ordinary least squares regressions due to the possibility that the error term ${\epsilon }^{*}$ may be autocorrelated and heteroscedastic [25, 63, 67]. To address this, we set the data into a time series format by using the order of the rank variable in place of time and subsequently computed autocorrelation and heteroscedasticity-consistent Newey-West variance–covariance matrices [63, 67].

## Results

### Regional deprivation and ranking of districts

**Fig. 1:**
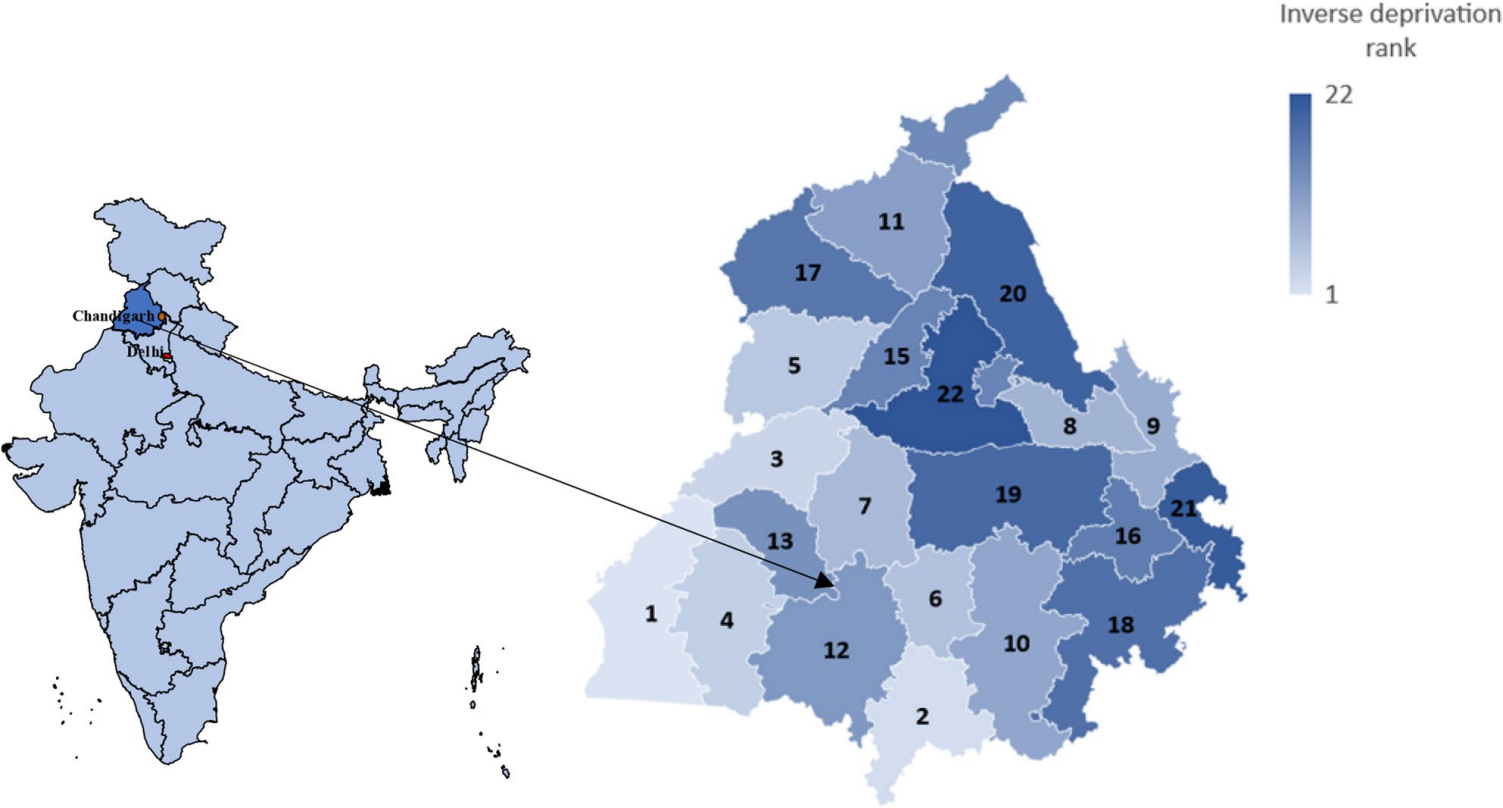
State-level map of India (left) and map of district-level deprivation in Punjab (right). Note: Areas marked in red are Chandigarh (the capital of Punjab and a union territory) and New Delhi (the capital of India). Darker shades in the Punjab map indicate lower levels of deprivation, lighter shades indicate higher levels of deprivation. All the maps were developed in Excel and Gramener

Table 2 presents the ranking of the 22 districts of Punjab by the PIMD, as well as the domain scores ${X}_{D}$ and the overall PIMD scores. The districts in the best-off quartile are Jalandhar, Sahibzada Ajit Singh Nagar, Hoshiarpur, Ludhiana, Patiala, and Amritsar, whereas Tarn Taran, Sri Muktsar Sahib, Firozpur, Mansa and Fazilka are in the most deprived quartile. The two worst-off districts, Fazilka and Mansa, both exhibit the highest domain score of 100, and thus the highest level of deprivation, each in two domains. The highest domain-specific deprivation score was observed in the worst-off PIMD quartile for 5 out of 6 domains. The only exception is the health deprivation domain, which has the highest score for Rupnagar in the second-worst PIMD quartile. The best-off end of the PIMD ranking seems to be less homogenous. Low scores in one or two domains are scattered across the better-off half, only Jalandhar has consistently low domain-specific deprivation scores in almost all domains. Note that we inverted the area deprivation rank and assigned the highest rank to the least deprived district to enable comparison of the results for area deprivation and wealth. Figure 1 shows a map of Punjab with the different colour brightness indicating the degree of deprivation according to the PIMD rank.

**Table 2:** Domain-specific and overall deprivation of districts

	Deprivation scores							Inverse deprivation rank
Districts	Economic	Infrastructure	Health	Living Environment	Healthcare	Education	Mean	
Fazilka	40.76	100	12.78	47.81	100	47.81	58.19	1 (worst-off)
Mansa	33.09	52.35	37.91	100	17.78	100	56.85	2
Firozpur	40.76	65.57	12.78	47.81	52.35	47.81	44.51	3
Sri Muktsar Sahib	22.75	37.91	29.1	33.09	65.57	65.57	42.33	4
Tarn Taran	52.35	44.01	10.23	65.57	22.75	33.09	38	5
Barnala	100	1.06	17.78	22.75	29.1	25.71	32.73	6
Moga	20.13	20.13	33.09	37.91	44.01	20.13	29.23	7
Shahid Bhagat Singh Nagar	65.57	5.84	20.13	13.69	37.91	13.69	26.14	8
Rupnagar	17.78	10.23	100	11.9	10.23	4.55	25.78	9
Sangrur	3.32	17.78	65.57	29.1	11.9	22.75	25.07	10
Gurdaspur	27.34	15.65	6.52	18.93	33.09	7.93	18.24	11
Bathinda	13.69	29.1	4.55	15.65	1.06	37.91	16.99	12
Faridkot	11.9	25.71	1.06	25.71	7.21	29.1	16.78	13
Pathankot	27.34	22.75	6.52	18.93	13.69	7.93	16.19	14
Kapurthala	7.21	3.32	44.01	4.55	25.71	11.9	16.12	15
Fatehgarh Sahib	4.55	8.67	52.35	8.67	5.84	10.23	15.05	16
Amritsar	10.23	33.09	3.32	5.84	4.55	17.78	12.47	17
Patiala	8.67	4.55	25.71	7.21	2.16	15.65	10.66	18
Ludhiana	1.06	13.69	15.65	1.06	20.13	5.84	9.57	19
Hoshiarpur	15.65	2.16	8.67	10.23	15.65	1.06	8.9	20
Sahibzada Ajit Singh Nagar	5.84	7.21	22.75	3.32	8.67	2.16	8.33	21
Jalandhar	2.16	11.9	2.16	2.16	3.32	3.32	4.17	22 (best-off)

The table presents the domain-specific scores and the overall ranks of districts by the PIMD

### Inequalities in health: Individual-level versus area-level socio-economic status

Table 3 presents the prevalence of diabetes, general obesity, and abdominal obesity, along with associated wealth-related and deprivation-related inequalities. The overall prevalence of diabetes in Punjab is 6.13% whereas the prevalence of general obesity and abdominal obesity in Punjab was 39.33% and 58.26%, respectively. The positive value of the concentration indices for wealth-related inequalities indicates that diabetes (C = 0.1595) and both types of obesity (C = 0.1187 & C = 0.0755) are concentrated among better-off individuals. We observe positive and significant values of the concentration indices for deprivation-related inequalities in diabetes (C = 0.0526), general obesity (C = 0.053) and abdominal obesity (0.0275), though their magnitudes are smaller than for wealth-related inequalities.

**Table 3:** Concentration index and sensitivity of the concentration index

	Mean/prevalence (SE)	Wealth-specific inequality ${C}_{1}$ (SE)	Deprivation-specific inequality ${C}_{2}$ (SE)	Rank sensitivity $\Delta C={C}_{2}-{C}_{1}$ (SE)
Wealth index	1,144,901	0.3211	0.0672	−0.2539
	(10,959)	(0.0014)	(0.0016)	(0.0019)
Diabetes	0.0613	0.1595	0.0526	−0.1069
	(0.0015)	(0.0113)	(0.0113)	(0.0144)
General Obesity	0.3933	0.1187	0.0530	−0.0657
	(0.0033)	(0.0056)	(0.0053)	(0.0072)
Abdominal obesity	0.5826	0.0755	0.0275	−0.0480
	(0.006)	(0.004)	(0.004)	(0.0049)

All estimates are significant at 1% level. $\Delta C<0$ indicates stronger wealth-related than deprivation-related inequality

The Gini index for the wealth index is 0.3211. The results for rank sensitivity ($\Delta C)$ indicate that household-level wealth-related health inequalities are significantly stronger than area-level deprivation-related health inequalities.

The matrices in Tables 4, 5, and 6 show the domain-specific concentration indices on the main diagonal. The off-diagonal elements showing rank sensitivity $(\Delta C)$ compare the inequalities in the column domains with the inequalities in the row domain, i.e., the column domain concentration index is subtracted from the row domain concentration index. It shows the effect of changing the socio-economic status or area deprivation indicator on the measured inequality. We observed significantly positive concentration indices with respect to economic, infrastructure, living environment, healthcare, and educational domains, reflecting a higher concentration of diabetes (see Table 4), general obesity (see Table 5) and abdominal obesity (see Table 6) in better-off districts, only the results for the health domain were non-significant in the case of diabetes. The estimated differences between the area-level deprivation-related concentration indices for diabetes indicate no statistically significant differences between the deprivation domains, indicating that ranking by separate domains instead of the overall PIMD would not necessarily yield significantly different results. The only exception is the health deprivation domain, in which no significant concentration in better-off or worse-off districts could be observed, such that all other domain-specific concentration indices suggest a significantly stronger concentration of diabetes in better-off districts.

**Table 4:** Matrix of domain-specific deprivation-related inequalities in diabetes and sensitivity analysis

	Deprivation domain						Overall PIMD (SE)	Household wealth index (SE)
	Economic (SE)	Infra-structural (SE)	Health (SE)	Living environment (SE)	Healthcare (SE)	Educational (SE)		
Economic deprivation domain	0.0624** (0.012)							
Infrastructural deprivation domain	0.0074 (0.0127)	0.0549** (0.1007)						
Health deprivation domain	0.0711** (0.0168)	0.0637** (0.0169)	−0.0088 (0.0115)					
Living environment deprivation domain	−0.0005 (0.0065)	−0.0079 (0.0118)	−0.0716** (0.0147)	0.0628** (0.0121)				
Healthcare deprivation domain	0.0191 (0.0139)	0.0117 (0.0135)	−0.0520** (0.0136)	0.0196 (0.0141)	0.0432** (0.0114)			
Educational deprivation domain	0.0054 (0.0109)	−0.002 (0.0082)	−0.0657** (0.0141)	0.0059 (0.0086)	−0.0137 (0.0151)	0.0569** (0.0107)		
Overall PIMD	0.0098 (0.008)	0.0024 (0.0096)	−0.0613* (0.0130)	0.0103 (0.006)	−0.0093 (0.0116)	0.0044 (0.0065)	0.0526** (0.0113)	
Household wealth index	−0.0971** (0.0152)	−0.1046** (0.0142)	−0.1682** (0.0157)	−0.0966** (0.0151)	−0.1163** (0.0155)	−0.1025** (0.0142)	−0.1069** (0.0144)	0.1595** (0.0113)

Concentration indices of diabetes with respect to domain score/wealth index on the main diagonal, off-diagonal elements comprise sensitivity analyses ($\Delta C={C}_{row}-{C}_{column}$), i.e., column element subtracted from row element; * indicates statistical significance at 5% level; **indicates statistical significance at 1% level

**Table 5:** Matrix of domain-specific deprivation-related inequalities in general obesity and sensitivity analysis

	Deprivation domain						Overall PIMD (SE)	Household wealth index (SE)
	Economic (SE)	Infra-structural (SE)	Health (SE)	Living environment (SE)	Healthcare (SE)	Educational (SE)		
Economic deprivation domain	0.0390** (0.0058)							
Infrastructural deprivation domain	0.0001 (0.0064)	0.0389** (0.0051)						
Health deprivation domain	0.0244* (0.0082)	0.0242** (0.0083)	0.0146* (0.0055)					
Living environment deprivation domain	−0.0148** (0.0033)	−0.0149* (0.006)	−0.0392** (0.007)	0.0538** (0.0059)				
Healthcare deprivation domain	0.0067 (0.0066)	0.0066 (0.0067)	−0.0177* (0.0063)	0.0215** (0.007)	0.0323** (0.0055)			
Educational deprivation domain	−0.0149* (0.0054)	−0.0151** (0.0038)	−0.0393** (0.007)	−0.0001 (0.0043)	−0.0216* (0.0073)	0.0539** (0.0053)		
Overall PIMD	−0.0139 (0.004)	−0.0141* (0.0047)	−0.0383** (0.0062)	0.0008 (0.0028)	−0.0207** (0.0056)	0.0010 (0.0031)	0.0530** (0.0053)	
Household wealth index	−0.0796 (0.0079)	−0.0798** (0.0071)	−0.1040** (0.0077)	−0.0649** (0.0078)	−0.0864** (0.0075)	−0.0647** (0.0072)	−0.0657** (0.0072)	0.1187** (0.0056)

Concentration indices of general obesity with respect to domain score/wealth index on the main diagonal, off-diagonal elements comprise sensitivity analyses ($\Delta C={C}_{row}-{C}_{column}$), i.e., column element subtracted from row element; * indicates statistical significance at 5% level; **indicates statistical significance at 1% level

**Table 6:** Matrix of domain-specific deprivation-related inequalities in abdominal obesity and sensitivity analysis

	Deprivation domain						Overall PIMD (SE)	Household wealth index (SE)
	Economic (SE)	Infra-structural (SE)	Health (SE)	Living environment (SE)	Healthcare (SE)	Educational (SE)		
Economic deprivation domain	0.0183** (0.004)							
Infrastructural deprivation domain	−0.0063 (0.0043)	0.0246** (0.0035)						
Health deprivation domain	0.0097* (0.0055)	0.0160 * (0.0057)	0.0086* (0.0038)					
Living environment deprivation domain	−0.0082** (0.0023)	−0.0019 (0.0041)	−0.0179** (0.0047)	0.0265** (0.004)				
Healthcare deprivation domain	−0.0003 (0.005)	0.0060 (0.0046)	−0.0100* (0.0042)	0.0079 (0.0047)	0.0186 ** (0.0038)			
Educational deprivation domain	−0.0110* (0.0036)	−0.0047 (0.0026)	−0.0207** (0.0047)	−0.0028 (0.0029)	−0.0107 (0.005)	0.0293** (0.0036)		
Overall PIMD	−0.0084* (0.0028)	−0.0021 (0.0031)	−0.0181** (0.0042)	−0.0003 (0.0019)	−0.0081 (0.0038)	0.0026 (0.002)	0.0275** (0.0038)	
Household wealth index	−0.0559** (0.0054)	−0.0496** (0.0048)	−0.0655* (0.0052)	−0.0477** (0.0053)	−0.0556** (0.0051)	−0.0449** (0.0049)	−0.0480** (0.005)	0.0755** (0.0038)

Concentration indices of abdominal obesity with respect to domain score/wealth index on the main diagonal, off-diagonal elements comprise sensitivity analyses ($\Delta C={C}_{row}-{C}_{column}$), i.e., column element subtracted from row element

^*^ indicates statistical significance at 5% level

^**^indicates statistical significance at 1% level

In the case of general and abdominal obesity, ranking by infrastructure deprivation instead of economic deprivation showed no effect on the health inequality estimate. In contrast, ranking by household-level wealth scores instead of the overall PIMD has a significant impact on C estimates (see Table 5 and Table 6), where ranking by household-level wealth consistently shows stronger concentration indices than ranking by PIMD or separate domains.

The bottom row in Tables 4, 5, and 6 comprises the differences between wealth-related health inequalities and the concentration indices with respect to domain-specific area-level deprivation. The significantly negative differences when comparing area deprivation-related inequalities to wealth-related inequality in the bottom line of each table suggest that individual-level inequality is more pronounced than area-level deprivation-related health inequality.

## Discussion

This paper is the first to construct an index of multiple deprivation for the Indian state of Punjab. It is also the first paper to investigate the association of diabetes and two of its risk factors with area deprivation as one of its several determinants in an Indian context. The PIMD ranking suggests significant geographical disparities, with Fazilka and Mansa exhibiting the highest deprivation scores. These districts also had the highest domain-specific deprivation scores, particularly in infrastructure, living environment, healthcare and education. Districts falling into the most deprived quartile, i.e. Fazilka, Firozpur, Mansa, and Muktsar, heavily depend on agriculture and tend to receive less investment in industrialization and infrastructure projects [44].

The results indicate a high prevalence of diabetes and two of its prominent risk factors, general obesity and abdominal obesity, in Punjab, which is a significant public health challenge. The findings also indicate that diabetes and these risk factors are more concentrated among the rich in Punjab, which is consistent with the existing literature on India as a whole [12, 13, 59], and that they are also concentrated in better-off districts. By definition, less deprived districts offer better facilities in terms of healthcare, education, infrastructure, and living environments. Nevertheless, the concentration of these health conditions in better-off regions and among better-off individuals persists. A potential explanation may be that the better-off in Punjab tend to live a sedentary lifestyle, whereas the poor are more often forced to be physically active [2, 26, 27]. Furthermore, the nutrition transition theory suggests that, with increased economic prosperity, people tend to adopt new dietary and lifestyle habits that can lead to a greater prevalence of non-communicable diseases related to nutrition [47]. One may speculate that a lower supply of fast-food options in more deprived areas may contribute to these findings, and that economic growth may have led to shifts in lifestyle choices, where individuals may engage in less physical activity. This effect might be reinforced when infrastructure and facilities are lacking, e.g., poor road connectivity and public transport, or when nearby schools are absent, requiring individuals to walk longer distances.

Another interesting finding was the stark difference in the prevalence of general obesity (BMI-based) and abdominal obesity. Asian Indians are more susceptible to abdominal obesity due to their tendency to deposit fat around their abdomen [24]. Abdominal obesity is on the rise in rural India, and is now penetrating lower and middle socio-economic groups as well. It could be due to a reduction in the share of agricultural activities and an increase in non-farm activities among the rural population [9], which also leads to a shift in dietary patterns. Often, BMI-based obesity measures are used in clinical and research settings; however, it does not account for adiposity [10]. Therefore, BMI-based obesity can underestimate the actual burden of obesity in the country.

Comparing inequalities measured using individual-level socio-economic status with those computed based on area-level indicators suggested that household-level wealth-related inequalities are significantly more pronounced than inequalities related to area deprivation. This difference is not unique to Punjab; Siegel et al., [56] made a similar observation for Germany. A technical explanation might be that the area-level deprivation-related inequality yielded fewer values for socio-economic status and thus may have missed the heterogeneities expected within districts. Nevertheless, this finding may also imply that individual-level determinants may have a stronger, more direct effect on diabetes and obesity than the broader area-level factors.

Like other studies, this research is also not free from limitations. The first limitation relates to the so-called modifiable areal unit problem [11, 17, 54, 62]. Districts were the smallest areal unit for which official statistics were available. We acknowledge that the exact ranking of individuals by the PIMD depends to some extent on the size and administrative boundaries of the districts, as potential heterogeneities within the districts remain unobserved. The second limitation arises from the creation of new districts in July 2011. In order to be able to include all 22 districts, we assigned the same values for the administrative variables taken from the Census 2011 [8] to Fazilka and Firozpur, and to Pathankot and Gurdaspur. A more precise distinction between the two districts would require new data, which were not available at the time of the study.

The third limitation is that our study uses indicators from different data sources and different time periods, which may introduce bias. However, in our analysis, each indicator was drawn from a single, consistent data source and time period across all districts. Therefore, while there may be temporal differences between indicators, there is no within-indicator variation across districts that could bias the comparative analysis. Moreover, since our objective was to capture the relative differences between districts rather than time trends, we believe this approach is appropriate and does not compromise the validity of our results.

The fourth limitation is that our study cannot distinguish between type 1 and type 2 diabetes. We argue that type 1 diabetes is mainly genetic and is unlikely to follow a socio-economic gradient and, therefore, is unlikely to bias our inequality indicators [55]. Fifth, we computed Gini indices for wealth inequality using an index based on principal component analysis. Although we acknowledge that this is a dimensionless indicator, comparing inequalities in the weighted score computed from the observed presence of different assets and facilities may still provide a valid idea of differences in levels of inequalities in living standards across different districts. Sixth, we included data on districts which were not part of our analysis (i.e., districts in Kerala, Tamil Nadu, Goa and Himachal Pradesh) in the factor analysis for the computation of the PIMD domain scores. Although we cannot rule out that this may have induced a small bias in the factor score weights when computing the deprivation domain scores, we argue that the included states are similar enough since they are still within the same country, belong to the highest Epidemiological Transitioning Levels like Punjab, and the all-age DALY rates for diabetes are also similarly high.

Finally, the weighting of the different domain scores in indices of multiple deprivation is always arbitrary to some extent. Different contexts may require one to consider different priorities, thus different weights, for the different deprivation domains. Our example uses the arithmetic mean of the domain scores, which implies an assumed equal relevance of the different domains. However, depending on the context, future applications of the index may require a refined weighting scheme for the domain scores. Additionally, it should also be noted that the findings are not interpreted as causal. Furthermore, while this analysis presents the construction of the PIMD and a first comparison of PIMD-related and household-level wealth-related inequalities, future analysis may include multilevel modelling to further disentangle PIMD-related district-level determinants of health from household-level wealth-related determinants of health.

## Conclusion

Unlike findings from HICs, diabetes, general obesity and abdominal obesity are concentrated in better-off areas and among better-off individuals in the Indian state of Punjab. Although the association between household-level wealth and health conditions is a bit stronger than between area-level deprivation and health, our findings suggest that area deprivation represents an important determinant of the prevalence and socio-economic gradients in diabetes and obesity. Compared to individual-level determinants, area-level information is easily available from administrative data and is more suitable for the planning of the allocation of resources for healthcare and prevention. Indices of multiple deprivation may therefore help policymakers to address the regionally unequal burden when allocating resources to prevent or mitigate the spread of diabetes and its risk factors in India. Specifically, the relatively better-off districts could be prioritized, with them receiving proportionally greater attention for prevention and control activities through the existing health system and community-based programs. The concentration of diabetes, general and abdominal obesity among better-off districts and households suggests that efforts should be made to increase awareness and promote healthy lifestyles, particularly in affluent areas. Public health campaigns and initiatives aimed at encouraging physical activity and reducing the consumption of unhealthy foods could be implemented in better-off areas and may be particularly effective when addressing economically better-off households.

## Supplementary Information

Supplementary Material 1

## Data Availability

Data is available in the public domain and can be retrieved from: http://www.dhsprogram.com.

## Abbreviations

HICs: High Income Countries
WHO: World Health Organization
LMICs: Low- and Middle-Income Countries
DALYs: Disability Adjusted Life Years
ICMR: Indian Council of Medical Research
PHFI: Public Health Foundation of India
IHME: Institute of Health Metrics and Evaluation
PIMD: Punjab Index of Multiple Deprivation
BPL: Below Poverty Line
BMI: Body Mass Index
IPHS: Indian Public Health Standards
NITI Aayog: National Institution for Transforming India Aayog
NFHS: National Family Health Survey
DISE: District Information System for Education.
RHS: Rural Health Statistics
PCA: Principal Component Analysis
GWPCA: Geographically Weighted Principal Component Analysis
ML: Maximum Likelihood
ETLs: Epidemiological Transitioning Levels
SE: Standard Error
