# Supplementary material for: Developing a Punjab Index of Multiple Deprivation to investigate regional health inequalities in North-Western India
Source: BMC Public Health. 2026 Jan 8;26:353. doi: 10.1186/s12889-025-26073-x (PMC12849119; doi:10.1186/s12889-025-26073-x)
Supplement: Supplementary file 1 — Supplementary Material 1 [file 12889_2025_26073_MOESM1_ESM.docx]

**Appendix**

1. **Sample distribution across different socioeconomic and demographic groups in Punjab**

| **Variable** | **N (weighted)** | **Percentage (%)** |
| --- | --- | --- |
| **Place of residence** | | |
| Urban | 19,128 | 36.3 |
| Rural | 33,554 | 63.7 |
| **Age group** | | |
| 15–29 | 17,180 | 32.6 |
| 30–39 | 11,070 | 21.0 |
| 40–59 | 15,105 | 28.7 |
| 60 and above | 9,327 | 17.7 |
| **Sex** | | |
| Male | 24,966 | 47.4 |
| Female | 27,716 | 52.6 |
| **Wealth index** | | |
| Poorest | 10,073 | 19.1 |
| Poorer | 10,575 | 20.1 |
| Middle | 10,592 | 20.1 |
| Richer | 10,847 | 20.6 |
| Richest | 10,595 | 20.1 |
| **Religion** | | |
| Hindu | 19,456 | 36.9 |
| Sikh | 31,719 | 60.2 |
| Others | 1,507 | 2.9 |
| **Social Category** | | |
| General | 20,621 | 39.1 |
| OBCs | 7,153 | 13.6 |
| SCs/STs | 23,724 | 45.0 |
| Don’t know | 1,184 | 2.3 |
| **Marital status** | | |
| Never married | 12,870 | 24.4 |
| Married | 35,132 | 66.7 |
| Widowed/Divorced/Deserted | 4,680 | 8.9 |
| **PIMD quintile** | | |
| 1 (most deprived) | 7,751 | 14.7 |
| 2 | 6,910 | 13.1 |
| 3 | 11,519 | 21.9 |
| 4 | 8,776 | 16.7 |
| 5 (least deprived) | 17,727 | 33.7 |
| **Total** | **52,682** | **100** |

OBC: Other Backwards Class, PIMD: Punjab Index of Multiple Deprivation, SCs: Scheduled castes, STs: Scheduled Tribes, Other religious category includes Muslims, Christians, Buddhists/Neo-Buddhists, Jains, Jews, Parsis/Zoroastrians, no religion and others.

1. **Sample distribution across districts of Punjab**

| **Districts** | **Number of individuals (weighted)** | **SE** | **LCL** | **UCL** |
| --- | --- | --- | --- | --- |
| Fazilka | 2561.37 | 75.29 | 2413.59 | 2709.16 |
| Mansa | 1587.74 | 52.55 | 1484.59 | 1690.90 |
| Firozpur | 1723.24 | 80.15 | 1565.92 | 1880.56 |
| Sri Muktsar Sahib | 1878.73 | 86.35 | 1709.23 | 2048.23 |
| Tarn Taran | 2041.96 | 92.27 | 1860.86 | 2223.07 |
| Barnala | 1216.63 | 58.68 | 1101.46 | 1331.81 |
| Moga | 2263.79 | 74.60 | 2117.36 | 2410.21 |
| Shahid Bhagat Singh Nagar | 1387.65 | 47.06 | 1295.27 | 1480.02 |
| Rupnagar | 1264.02 | 51.31 | 1163.30 | 1364.74 |
| Sangrur | 3198.43 | 137.07 | 2929.37 | 3467.48 |
| Gurdaspur | 2948.17 | 191.12 | 2573.03 | 3323.31 |
| Bathinda | 2784.16 | 202.49 | 2386.72 | 3181.61 |
| Faridkot | 1324.14 | 34.11 | 1257.18 | 1391.10 |
| Pathankot | 1279.63 | 68.06 | 1146.04 | 1413.22 |
| Kapurthala | 1746.90 | 60.22 | 1628.69 | 1865.10 |
| Fatehgarh Sahib | 1015.57 | 54.93 | 907.75 | 1123.39 |
| Amritsar | 4733.37 | 164.49 | 4410.50 | 5056.23 |
| Patiala | 3191.02 | 220.92 | 2757.38 | 3624.66 |
| Ludhiana | 5287.52 | 353.02 | 4594.59 | 5980.44 |
| Hoshiarpur | 3694.16 | 87.76 | 3521.90 | 3866.42 |
| Sahibzada Ajit Singh Nagar | 1751.86 | 116.01 | 1524.16 | 1979.56 |
| Jalandhar | 3801.95 | 187.34 | 3434.24 | 4169.66 |
| **Punjab** | **52,682** | **640.45** | **51424.89** | **53939.11** |

Note: N is the weighted sample size; SE: standard error; LCL: 95%-lower confidence level; UCL: 95%-upper confidence level

1. **Sample distribution across additional districts**

|  | **Number of individuals (weighted)** | **SE** | **LCL** | **UCL** |
| --- | --- | --- | --- | --- |
| **Himachal Pradesh** | **28,572** | **510.7** | **27570.2** | **29573.1** |
| Chamba | 1949 | 68.6 | 1813.7 | 2083.3 |
| Kangra | 6432 | 258.0 | 5924.7 | 6938.8 |
| Lahul & spiti | 102 | 4.2 | 93.9 | 110.3 |
| Kullu | 1732 | 61.6 | 1610.5 | 1852.7 |
| Mandi | 4372 | 271.5 | 3838.7 | 4905.8 |
| Hamirpur | 1764 | 52.8 | 1660.3 | 1867.9 |
| Una | 2012 | 62.8 | 1888.3 | 2135.0 |
| Bilaspur | 1581 | 65.7 | 1452.2 | 1710.3 |
| Solan | 2369 | 104.5 | 2163.4 | 2574.0 |
| Sirmaur | 2167 | 83.2 | 2003.2 | 2330.3 |
| Shimla | 3745 | 288.0 | 3178.6 | 4310.6 |
| Kinnaur | 349 | 14.7 | 319.6 | 377.5 |
| **Goa** | **5,220** | **222.2** | **4784.4** | **5655.6** |
| North goa | 2874 | 196.8 | 2481.5 | 3265.5 |
| South goa | 2347 | 103.0 | 2141.3 | 2551.7 |
| **Kerala** | **32,330** | **350.9** | **31641.7** | **33017.9** |
| Kasaragod | 1391 | 64.3 | 1264.7 | 1517.1 |
| Kannur | 2442 | 69.8 | 2305.3 | 2579.6 |
| Wayanad | 832 | 44.4 | 745.2 | 919.8 |
| Kozhikode | 3086 | 98.5 | 2892.6 | 3279.6 |
| Malappuram | 3957 | 131.6 | 3698.8 | 4215.8 |
| Palakkad | 2880 | 107.8 | 2668.2 | 3091.9 |
| Thrissur | 2868 | 116.9 | 2638.2 | 3097.7 |
| Ernakulam | 3414 | 121.4 | 3175.5 | 3652.3 |
| Idukki | 1082 | 26.5 | 1029.6 | 1133.8 |
| Kottayam | 1969 | 93.4 | 1785.4 | 2152.4 |
| Alappuzha | 2274 | 76.9 | 2123.1 | 2425.3 |
| Pathanamthitta | 1115 | 35.9 | 1044.2 | 1185.3 |
| Kollam | 2410 | 74.6 | 2263.7 | 2556.6 |
| Thiruvananthapuram | 2609 | 151.4 | 2311.4 | 2906.4 |
| **Tamil nadu** | **68,825** | **1005.4** | **66853.3** | **70795.8** |
| Thiruvallur | 3231 | 211.5 | 2816.5 | 3646.5 |
| Chennai | 3656 | 295.4 | 3076.1 | 4235.3 |
| Kancheepuram | 3580 | 188.4 | 3210.1 | 3949.5 |
| Vellore | 3632 | 183.5 | 3272.0 | 3991.9 |
| Tiruvannamalai | 2309 | 88.6 | 2134.6 | 2482.4 |
| Viluppuram | 2982 | 146.8 | 2693.7 | 3269.8 |
| Salem | 3399 | 123.3 | 3157.1 | 3640.9 |
| Namakkal | 1593 | 53.7 | 1487.5 | 1698.2 |
| Erode | 2890 | 727.4 | 1462.9 | 4317.1 |
| The nilgiris | 856 | 54.6 | 749.2 | 963.2 |
| Dindigul | 2493 | 106.7 | 2284.0 | 2702.5 |
| Karur | 895 | 39.1 | 817.7 | 971.3 |
| Tiruchirappalli | 2584 | 110.9 | 2366.1 | 2801.1 |
| Perambalur | 510 | 15.1 | 480.3 | 539.6 |
| Ariyalur | 687 | 25.9 | 635.8 | 737.5 |
| Cuddalore | 2634 | 93.0 | 2451.7 | 2816.7 |
| Nagapattinam | 1771 | 145.6 | 1485.1 | 2056.5 |
| Thiruvarur | 1109 | 37.4 | 1035.2 | 1181.9 |
| Thanjavur | 1721 | 81.4 | 1561.6 | 1881.1 |
| Pudukkottai | 1437 | 93.8 | 1252.8 | 1620.7 |
| Sivaganga | 1306 | 87.4 | 1134.9 | 1477.7 |
| Madurai | 3208 | 136.8 | 2939.5 | 3476.2 |
| Theni | 1326 | 56.1 | 1215.6 | 1435.7 |
| Virudhunagar | 2001 | 65.0 | 1873.3 | 2128.4 |
| Ramanathapuram | 1381 | 56.7 | 1270.1 | 1492.5 |
| Thoothukkudi | 1945 | 118.7 | 1711.9 | 2177.5 |
| Tirunelveli | 3105 | 116.1 | 2877.5 | 3333.2 |
| Kanniyakumari | 1555 | 94.6 | 1369.6 | 1740.9 |
| Dharmapuri | 1565 | 108.9 | 1351.5 | 1778.7 |
| Krishnagiri | 1696 | 67.6 | 1563.0 | 1828.4 |
| Coimbatore | 3287 | 184.2 | 2925.8 | 3648.5 |
| Tiruppur | 2483 | 182.9 | 2123.7 | 2841.4 |

Note: N is the weighted sample size; SE: standard error; LCL: 95%-lower confidence level; UCL: 95%-upper confidence level

1. **Performance of selected Indian states on several health indicators**

Source: NFHS-5

Source: Sujata et al., 2024

Source: Dandona et al., 2017
